# Comparison of triple-negative breast cancer molecular subtyping using RNA from matched fresh-frozen versus formalin-fixed paraffin-embedded tissue

**DOI:** 10.1186/s12885-017-3237-1

**Published:** 2017-04-04

**Authors:** Bojana Jovanović, Quanhu Sheng, Robert S. Seitz, Kasey D. Lawrence, Stephan W. Morris, Lance R. Thomas, David R. Hout, Brock L. Schweitzer, Yan Guo, Jennifer A. Pietenpol, Brian D. Lehmann

**Affiliations:** 1 Medical Oncology Department, Dana-Farber Cancer Institute, Harvard Medical School and Broad Institute, Boston, 02215 MA USA; 2 Center for Quantitative Sciences, Vanderbilt University School of Medicine, Nashville, 37232 TN USA; 3 Insight Genetics Incorporated, Nashville, 37217 TN USA; 4 Department of Biochemistry, Vanderbilt University, Nashville, 37232 TN USA; 5 Vanderbilt-Ingram Cancer Center, Vanderbilt University Medical Center, Nashville, TN 37232 USA

**Keywords:** TNBCtype, Fresh-frozen, Formalin-fixed paraffin embedded, RNA-seq

## Abstract

**Background:**

Triple negative breast cancer (TNBC) is a heterogeneous disease that lacks unifying molecular alterations that can guide therapy decisions. We previously identified distinct molecular subtypes of TNBC (TNBCtype) using gene expression data generated on a microarray platform using frozen tumor specimens. Tumors and cell lines representing the identified subtypes have distinct enrichment in biologically relevant transcripts with differing sensitivity to standard chemotherapies and targeted agents. Since our initial discoveries, RNA-sequencing (RNA-seq) has evolved as a sensitive and quantitative tool to measure transcript abundance.

**Methods:**

To demonstrate that TNBC subtypes were similar between platforms, we compared gene expression from matched specimens profiled by both microarray and RNA-seq from The Cancer Genome Atlas (TCGA). In the clinical care of patients with TNBC, tumor specimens collected for diagnostic purposes are processed by formalin fixation and paraffin-embedding (FFPE). Thus, for TNBCtype to eventually have broad and practical clinical utility we performed RNA-seq gene expression and molecular classification comparison between fresh-frozen (FF) and FFPE tumor specimens.

**Results:**

Analysis of TCGA showed consistent subtype calls between 91% of evaluable samples demonstrating conservation of TNBC subtypes across microarray and RNA-seq platforms. We compared RNA-seq performed on 21-paired FF and FFPE TNBC specimens and evaluated genome alignment, transcript coverage, differential transcript enrichment and concordance of TNBC molecular subtype calls. We demonstrate that subtype accuracy between matched FF and FFPE samples increases with sequencing depth and correlation strength to an individual TNBC subtype.

****Conclusion**s:**

TNBC subtypes were reliably identified from FFPE samples, with highest accuracy if the samples were less than 4 years old and reproducible subtyping increased with sequencing depth. To reproducibly subtype tumors using gene expression, it is critical to select genes that do not vary due to platform type, tissue processing or RNA isolation method. The majority of differentially expressed transcripts between matched FF and FFPE samples could be attributed to transcripts selected for by RNA enrichment method. While differentially expressed transcripts did not impact TNBC subtyping, they will provide guidance on determining which transcripts to avoid when implementing a gene set size reduction strategy.

**Trial registration:**

NCT00930930 07/01/2009.

**Electronic supplementary material:**

The online version of this article (doi:10.1186/s12885-017-3237-1) contains supplementary material, which is available to authorized users.

## Background

Multi-gene prognostic and predictive gene signatures are now routinely performed in pathology laboratories to guide treatment decisions in breast [1], lung [2] and colorectal cancers [3]. Previously we identified transcriptional heterogeneity and unique molecular subtypes from patients with triple negative breast cancer (TNBC) using data from gene expression microarrays [4]. The TNBC molecular subtypes identified include two basal-like (BL1 and BL2), an immunomodulatory (IM), a mesenchymal (M), a mesenchymal stem-like (MSL), and a luminal AR (LAR) subtype [4]. Retrospective analysis of TNBC molecular subtypes was shown to be predictive of response to neoadjuvant anthracycline and cyclophosphamide followed by taxane [5]. Our previous study showed BL1 had the highest pCR rate (50%) at time of surgery and BL2 and LAR had the lowest (0% and 10%, respectively) [5]. The ability to determine TNBC molecular subtyping may guide treatment decisions for TNBC patients.

Originally, TNBC molecular subtypes were identified by correlating gene expression to six centroids comprising 2188 genes representing each subtype [4]. Recently, these subtypes were further reduced into four tumor intrinsic subtypes by the removal of the immunomodulatory and mesenchymal-stem like subtypes due to confounding expression from infiltrating immune and surrounding stromal cells, respectively [6]. For clinical utility, large gene signatures require size reduction and need to be generated using RNA isolated from routine FFPE tissues. Reproducible subtyping requires careful selection of genes that do not vary by platform, tissue processing or RNA isolation methods. Genes that are preferentially degraded in FFPE or inadequately selected for by poly (A) selection should be avoided when selecting smaller panels of genes.

Transcriptional profiling by RNA sequencing (RNA-seq) provides unprecedented sensitivity and quantitative measurement of a diverse range of RNA species [7]. However, transcriptome composition will differ depending on enrichment protocols. In order to remove the overwhelming majority of ribosomal RNA (rRNA) transcripts, RNA can be enriched by poly (A) selection or ribosomal depletion. Poly (A) enrichment fails to capture non-poly (A) transcripts or those that are partially degraded and is not compatible with RNA isolated from formalin-fixed paraffin-embedded (FFPE) tissues. Fresh-frozen (FF) tissue is ideal for all methods of transcriptional quantification, however they are not widely available for routine clinical assays and are costly to collect and maintain. In contrast, FFPE tissue samples are routinely collected throughout patient care and yield relatively low amounts of degraded RNA. Several studies have demonstrated that RNA-seq using FFPE provides reliable gene expression profiling correlating well with FF tissue analyzed by quantitative PCR [2, 8–11].

In order to determine if RNA-seq would produce high quality data and permit proper molecular classification of TNBC, we compared the gene expression and TNBC subtype concordance with RNA obtained from 98 matched FF and FFPE tumors processed on microarray or RNA-seq platforms, respectively. The feasibility of performing molecular subtyping on RNA isolated from TNBC specimens was further determined by comparing RNA-seq data from 21 matched FF and FFPE samples sequenced on either Illumina HiSeq or MiSeq platforms. Our analyses show that the majority of differential transcripts identified between matched FF and FFPE samples could be attributed to RNA isolation methods.

## Methods

### Tumor tissue acquisition

Tumor tissues were acquired from an active clinical trial and the Vanderbilt Cooperative Human Tissue Network (CHTN). All patients provided written informed consent prior to enrollment and were approved by an Independent Ethics Committee at Vanderbilt University. Tissues from the CHTN have been obtained from de-identified female patients. Vanderbilt University Institutional Review Board (exempt form IRB#090026) was reviewed and determined that the study does not qualify as “human subject” research per §46.102(f) (2). Tissue was obtained from the pathology lab before being discarded. No identifiers were included and there was no contact with donor.

### FF RNA isolation and poly (a) capture

FF tissue sections were processed using the RNeasy Mini Kit (QIAGEN cat. # 74104). Briefly, the samples were disrupted in 350 μL Buffer RLT with dithiothreitol (DTT), and 0.5 mm glass beads (BioSpec cat # 11079105) on a Mini-Beadbeater (Bio-Spec cat # 3110BX). The tissue lysate was then purified via RNAeasy column and eluted in 50 μL nuclease-free water. RNA quantity was measured via Qubit RNA assay (Life Technologies cat # Q32855) and quality via Agilent 2200 TapeStation R6K ScreenTape assay (cat # 5067–5367).

FF samples were enriched for messenger RNA (mRNA) by treating the total RNA (tRNA) extracted from tissue sections with NEBNext Magnetic Oligo d (T)25 Beads (New England Biolabs cat. # E7490S) that bind to the poly-A tail of mRNA molecules. After repeated washes and re-suspension in binding buffer, the NEBNext beads were combined with tRNA and incubated at 65 °C for 5 min on a thermal cycler before bring the temperature down to 4 °C in order to denature the RNA and facilitate binding of the poly-A tailed RNA to the beads. When the samples reached 4 °C they were removed from the thermal cycler and allowed to incubate at room temperature for 5 min before being placed on a magnetic rack for 2 min to separate the poly-A RNA bound to the beads from the solution. After removal of the supernatant, the samples were washed twice to remove unbound RNA. The samples were then placed back on the magnet and the supernatant was removed after a 2 min incubation at room temperature. The beads were then re-suspended in the Tris buffer and mixed thoroughly by pipette before being placed on a thermal cycler and incubated at 80 °C for 2 min and then cooled 25 °C to elute the poly-A RNA from the beads. Tubes were removed from the thermal cycler when the temperature reached 25 °C. Binding buffer was added to each sample to facilitate the RNA’s binding to the beads and they were incubated at room temperature for 5 min before being placed on the magnetic stand. After 2 min on the magnet the supernatant was removed and discarded. The beads were then rinsed twice with Wash Buffer while off the magnet. The mRNA was then eluted off the beads by adding 17 μl of the Tris Buffer and incubating the sample at 80 °C for 2 min before immediately returning the samples to the magnet. After 2 min on the magnet the purified mRNA was collected by transferring the supernatant to a clean nuclease-free PCR tube and placing on ice.

### FFPE RNA isolation and rRNA reduction

FFPE tissue sections were deparaffinized using deparaffinization solution (QIAGEN cat # 19093) processed with the RNeasy FFPE kit (QIAGEN cat # 73504). FFPE RNA was eluted in 25 μL nuclease-free water. RNA quantity was measured via Qubit RNA assay and quality via Agilent 2200 TapeStation R6K ScreenTape assay (cat # 5067–5367. Due to the lack of RIN numbers for FFPE samples, the RNA size was qualified by an average base pair fragment size ranging from 65 to 1000 bp, and recorded. For FFPE samples, the ribosomal RNA was removed from the samples using the Ribo-Zero Gold (H/M/R) - Low input (Epicentre cat # LIG1224) kit. This was only performed on FFPE samples where an adequate amount of RNA was extracted from the tissue (0.2–1.0 μg of RNA). Samples with less than 0.2 μg of RNA were not rRNA depleted but treated with the TotalScript library preparation protocol described below rather than the ScriptSeq protocol used on all other samples. Prior to beginning rRNA depletion, the Ribo-Zero beads were removed from the storage solution and washed twice with RNase-Free water (before being resuspended in Magnetic Bead Resuspension Solution and combined with RiboGuard RNase Inhibitor) and stored at room temperature until needed. Next, the total RNA samples were combined with Ribo-Zero Reaction Buffer and Ribo-Zero rRNA Removal Solution and incubated at 68 °C for 10 min before a 5 min incubation at room temperature. This sample mixture was then combined with the washed magnetic beads that were prepared first and mixed thoroughly. After a 5 min incubation at room temperature, the samples were vortexed at level 5 for 10 s and placed at 50 °C for 5 min. The samples were then placed on the magnetic stand for 1 min before the supernatant (containing the ribosomal depleted RNA) was removed and transferred to a labeled RNase-Free microcentrifuge tube and purified using RNAClean XP (Beckman Coulter Cat. No. A63987).

### Library construction and sequencing

For FF samples, the mRNA isolated using the NEBNext PolyA Module was incubated at 85 °C for 5 min with random-hexamer primers linked to capture tags in fragmentation solution and then immediately placed on ice to simultaneously fragment the RNA anneal tagged cDNA synthesis primers. FFPE samples were not fragmented but were instead annealed to the cDNA synthesis primers by incubating the ribosomal RNA-depleted samples with tagged random hexamers at 65 °C for 5 min before placing them immediately on ice. For FFPE samples, the fragmentation solution was then added along with a cDNA synthesis master-mix and DTT. For FF samples, only the cDNA synthesis master-mix and DTT were added. cDNA synthesis was then performed on both sample types by incubating at 25 °C for 5 min followed by 42 °C for 20 min and then the temperature was raised to 37 °C and a Finishing Solution was added. The samples were then incubated at 37 °C for 10 min followed by 95 °C for 3 min before the temperature was reduced to 25 °C and random hexamers linked to a terminal tag and a DNA polymerase were added to apply a capture tag to the 3' end of the cDNA molecules. The samples were then incubated at 25 °C for 15 min and then 95 °C for 3 min before being placed immediately on ice. FFPE samples were then purified using the MinElute PCR Purification Kit from Qiagen. FF samples were purified using the Agencourt AMPure XP system from BeckmanCoulter at a bead to sample volume ratio of 0.9. All samples were then amplified by PCR using primers that anneal to the capture tags at the 3′ and 5′ ends of the cDNA. These sequencing primers also contained Illumina adapter sequences and sample identifier indexes, enabling the PCR to simultaneously amplify the cDNA library while also applying sequencing adaptors and indexes. After PCR, all samples were purified using the Agencourt AMPure XP system from BeckmanCoulter at a bead to sample volume ratio of 0.9 and quality was assessed using the Agilent 2200 TapeStation D1000 ScreenTape assay. The libraries were then quantified using the Library Quantification Kit from Kapa and diluted to a concentration of 4 nM. Samples analyzed on the same sequencer run were pooled equally and denatured by incubating in 0.2 N NaOH for five min before being diluted to 15 pM and sequenced on an illumina MiSeq instrument using version 3150 cycle kit. All sequences generated are deposited in the Sequence Read Archive under SRP101418.

### Sequencing alignment

Raw fastq files were obtained for each sample and aligned to the *Homo sapiens* reference using star [12]. The aligned reads were sorted and indexed using SAMtools and count tables were generated for each gene or exon using the HTSeq-count package [13].

### Gene expression and pathway evaluation

Differential genes between FF and FFPE (FDR *p*-value <0.5, absolute fold change >2 and average read count >4) were identified using DESeq2 [14]. Differential genes were analyzed by gene set enrichment analysis (GSEA) using cellular compartments (C5) and canonical pathways (C2, [15]).

### TNBC subtyping of gene expression from TCGA samples processed on microarray and RNA-seq platforms

RNA-seq and Agilent microarray gene expression data for TCGA breast cancer (BRCA) study were obtained from the Broad GDAC Firehose (http://gdac.broadinstitute.org/). Gene level 3 RSEM mRNA (stddata__2016_01_28 run) and lowess normalized Agilent microarray expression (stddata__2016_01_28 run) were downloaded and overlapping TNBC samples were identified by common participant barcode (TCGA-XX-XXX). RNA-seq and microarray data were subtyped independently as previously described [16].

### TNBC subtyping of matched FF and FFPE RNA-seq specimens

TNBC subtyping was performed on FPKM normalized RNA-seq counts from paired FF and FFPE samples uploaded in separate batches to TNBCtype website (http://cbc.mc.vanderbilt.edu/tnbc/) as previously described [16].

### Statistical methods

Spearman rank correlation coefficient was calculated on gene count for all FF/FFPE or HiSeq/MiSeq paired samples. The significance of correlation difference was performed by Mann-Whitney U test. Clopper-Pearson exact confidence bound was used to determine confidence interval for a binomial distribution.

## Results

### TNBC subtype ‘calls’ are highly concordant irrespective of data generation platform

To determine if TNBC subtypes that were previously identified from a meta-analysis of gene expression from publically available microarray data could be reliably reproduced with RNA-seq, we evaluated gene expression data generated from FF TNBC samples available from The Cancer Genome Atlas (TCGA). We identified 98 samples with gene expression data generated on both microarray and Illumina RNA-seq platforms. We performed TNBC subtyping and achieved a similar distribution of samples in TNBC subtypes as previously reported [4, 17] with 31% BL1, 18% BL2, 29% M, 18% LAR and 4% unclassified (Additional file 1: Table S1). The concordance between microarray and RNA-seq profiles was 91% [CI = 83.3%, 95.7%] (89 of 98 identical calls), with similar agreement to previous comparisons of prognostic signatures between platforms [4, 18], demonstrating conservation of TNBC subtypes across platforms (Additional file 1: Table S1). Since each sample receives a subtype correlation to one of the four subtype centroids, we compared the correlation of each sample to each of the subtypes for the microarray and RNA-seq data. There was a high degree of subtype correlation to each of the subtypes regardless of subtype call (R^2^: BL1 = 0.87, BL2 = 0.93, M = 0.97 and LAR = 0.83), demonstrating conservation of gene expression across RNA-seq and microarray platforms and suggesting that subtype discordance were likely due to other factors (Additional file 2: Fig. S1).

**Fig. 1:**
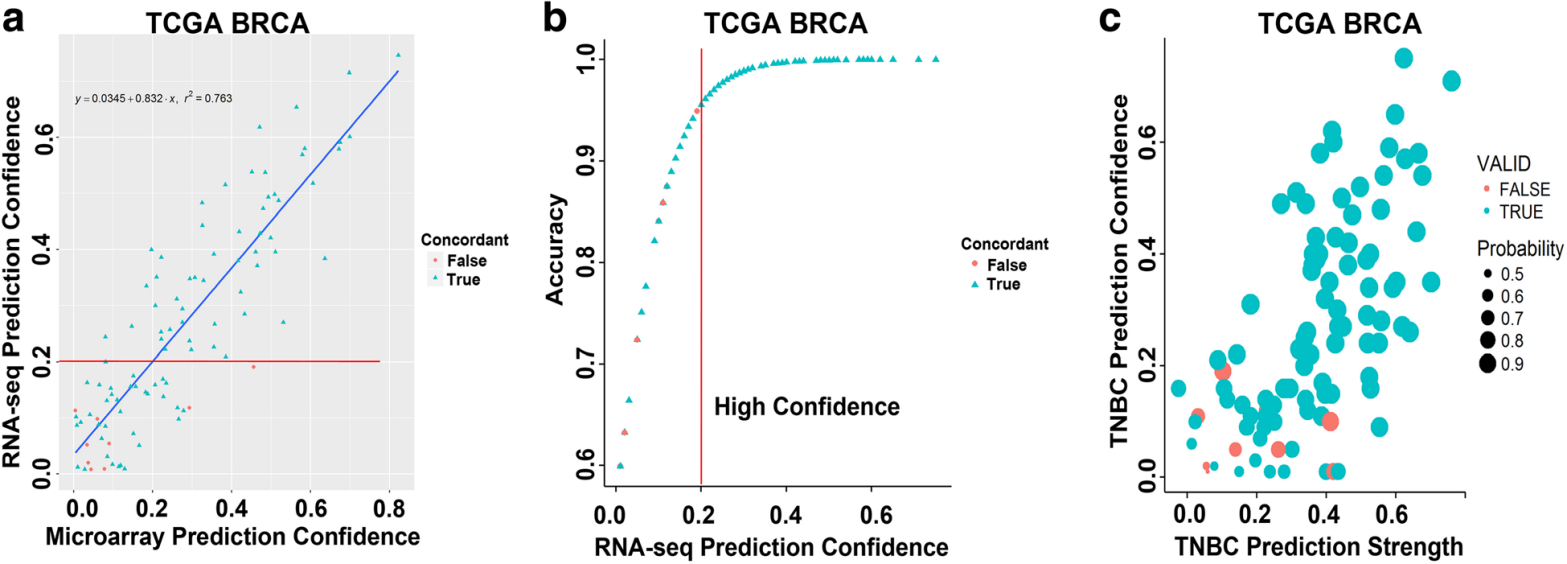
TNBC molecular subtype concordance between matched FF and FFPE samples processed on microarray and RNA-seq improves with increased prediction confidence. **a** *Scatterplot* shows TNBC subtype accuracy between microarray and RNA-seq as a function of prediction confidence in the TCGA breast (BRCA) cohort. **b** *Plot* shows RNA-seq prediction accuracy by confidence score. *Vertical line* cutoff demarks the prediction confidence score generating 95% concordance between platforms. **c** *Scatterplot* shows the concordance between microarray and RNA-seq platforms by strength of correlation to a subtype (prediction score)

Since TNBC subtype calls are generated from a continuous scale and can positively correlate to multiple subtypes as previously seen with overlapping PAM50 subtype distributions [19], we created a subtype prediction confidence score using the difference in correlation from the first and second highest subtype correlations Prediction confidence scores from matched microarray versus RNA-seq samples were highly correlated (r^2^ = 0.763) and discordant subtype calls were typically of low confidence (Fig. 1a). To determine an accurate confidence cutoff, we plotted the validation rate as a function of confidence score for RNA-seq platform (Fig. 1b). At a difference of 0.2 between the highest and second highest correlation, the prediction confidence was greater than 95% between matched microarray and RNA-seq samples (Fig. 1b). In addition, the prediction accuracy proportionally increased with the correlation strength of each subtype (Fig. 1c). These data demonstrate that TNBC subtype “calls” are highly reproducible between microarray and RNA-seq platforms when using FF tissue, especially when samples have high correlations to a single subtype.

### Comparison of RNA-seq performed on matched FF and FFPE TNBC samples

Since the majority of biopsy specimens clinically available, as part of routine care, are in the form of FFPE tissues, we evaluated the feasibility, robustness and reproducibility of molecular subtyping of FFPE-processed TNBC specimens. We collected matched FF and FFPE tumor RNA from 21 TNBC patients. We performed RNA-seq on total RNA enriched for the coding transcriptome, (poly-A mRNA selection) for FF tissues or ribosomal depleted RNA for FFPE, and sequenced 10 matched pairs of RNA using the Illumina MiSeq platform and 11 matched pairs of RNA using the Illumina HiSeq platforms (Additional files 1 and 3: Tables S1 and S2). Comparisons between platforms and tissue processing were made to evaluate genome alignment, transcript coverage, differential transcripts and concordance in TNBC molecular subtype calls.

### Genome alignment

Raw reads were aligned to the reference genome (hg19) with Ensembl (v75) annotation and read alignment was evaluated between platforms and tissue processing. The total number of aligned reads was similar between matched FF and FFPE samples on the MiSeq (1.30E^7^ vs. 1.36E^7^, *p* = 0.63) or HiSeq (8.08E^7^ vs. 8.53E^7^, *p* = 0.55) platforms, but the HiSeq had on average 6.2 times more aligned reads (Additional file 4: Fig. S2A and B). The number of aligned reads to the reference genome tended to vary by sample rather than sample processing and RNA isolation method, as demonstrated by lack of significant difference in the percentage of unmapped reads between FF and FFPE (8.8% vs. 7.6%, *p* = 0.5006) (Additional file 4: Fig. S2C). However, the number of unmapped reads was significantly increased in older (>10 years) FFPE samples compared to age matched FF samples (13% vs. 4%, *p* = 0.0066) (Additional file 4: Fig. S2D).

Since >80% of total RNA is composed of ribosomal RNA (rRNA), and 3′ RNA degradation is common in FFPE samples, poly-A transcript selection is not appropriate and ribosomal depletion provides a more accurate transcript estimation for FFPE samples. To determine the efficiency of ribosomal depletion, we calculated the ratio of rRNA to total coding RNA transcripts. The fraction of rRNA was less than 0.0011% and 0.0014% for poly-A selected transcripts from FF tissue processed on the MiSeq and HiSeq platforms, respectively. This fraction was similar to those obtained by ribosomal depleted RNA from FFPE on the MiSeq and HiSeq platforms (0.2169% and 0.0085%, respectively), demonstrating effective reduction of rRNA species.

### Transcriptome coverage

**Fig. 2:**
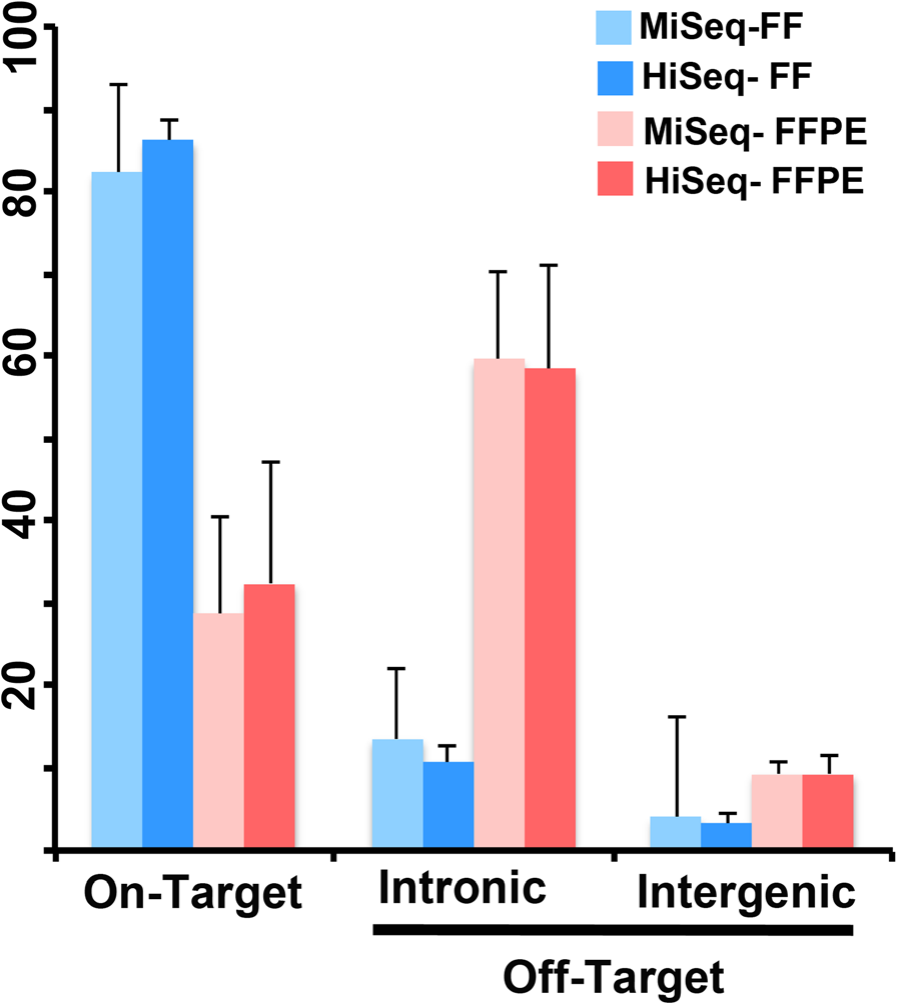
MiSeq and HiSeq platform mapped read comparison from FF- and FFPE-derived RNA sequences. **a** *Barplot* depicts the percentage of mapped reads that are on-target, or off-target (intronic and intergenic) for FF and FFPE samples processed on MiSeq and HiSeq platforms. **b** *Beeswarm box plot* shows mapped reads (%) form individual FF (*blue*) and FFPE (*red*) samples processed on the HiSeq

To determine the accuracy of transcript coverage, we evaluated the on- and off- target reads in each of the paired FFPE and FF samples. There were no significant differences between on-target, intergenic and intronic aligned reads on the MiSeq compared to the HiSeq platform (Fig. 2a). However, poly-A capture resulted in significantly more reads aligned to the transcriptome in the RNA isolated from FF specimens compared to FFPE specimens analyzed on both the MiSeq (82% vs. 28%, *p* < 0.0001) and HiSeq (86% vs. 34%, *p* < 0.0001) platforms (Fig. 2a). The vast majority of off-target reads enriched in FFPE samples were aligned to intronic regions, representing nascent unprocessed RNA species removed from FF samples by poly-A selection (HiSeq 60% vs. 14%, *p* < 0.0001 and MiSeq 58.4% vs. 10.7%, *p* < 0.0001) (Fig. 2a and b). In addition there were significantly more reads mapping to intergenic regions in FFPE compared to FF on both the HiSeq (8% vs. 3%, *p* = 0.001) and MiSeq (9% vs. 3%, *p* = 0.0187) platforms. The latter could be mitigated by increased coverage as the percentage of intergenic reads was decreased on the HiSeq platform.

### Correlation between FF and FFPE transcriptome profile

**Fig. 3:**
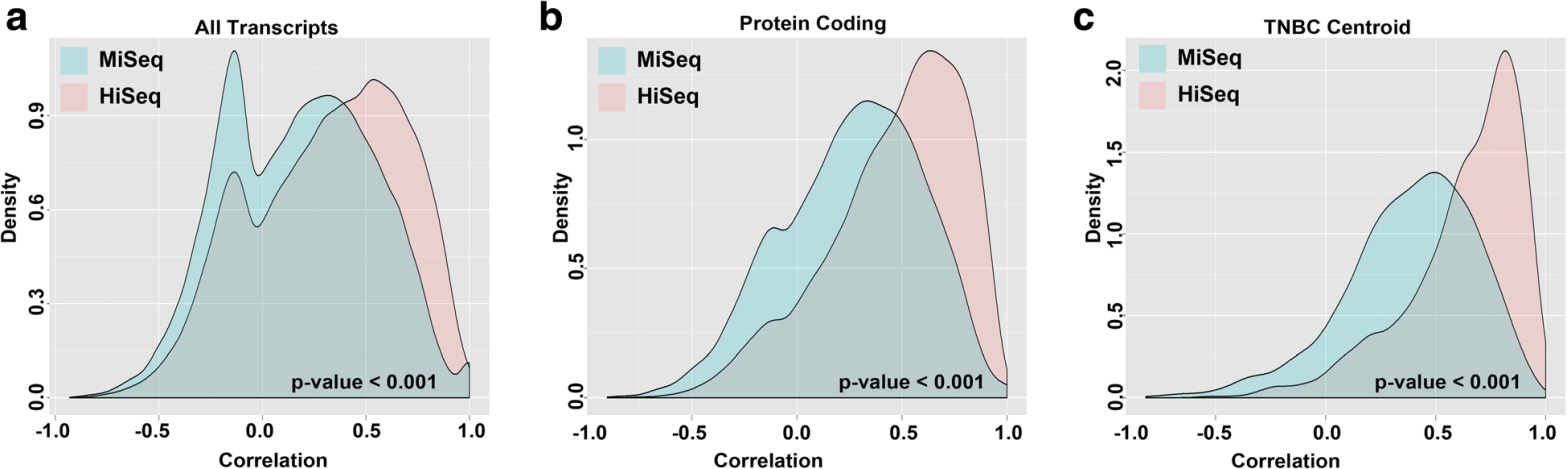
FF and FFPE transcript correlation improves with increased sequencing depth. *Density plots* show the pairwise Spearman correlation of matched FF and FFPE samples for **a** all transcripts, **b** protein-coding transcripts or **c** TNBC centroid transcripts processed on the HiSeq or MiSeq platform

Since the major difference between the MiSeq and HiSeq platforms is the number of total reads sequenced, we examined if this difference in coverage impacts overall transcript correlation between matched FF and FFPE samples. The correlation between matched FF and FFPE pairs were significantly (*p* < 0.0001) better on the HiSeq platform compared to the MiSeq platform, for all transcripts, protein-coding transcripts and those belonging to the TNBC centroid used to define TNBC subtypes, suggesting that MiSeq platform may not be sufficient for accurate transcript abundance estimation (Fig. 3).

The average Spearman correlation of gene expression from paired FF and FFPE samples was higher for HiSeq (0.88 +/− 0.04) samples compared to MiSeq (0.83 +/− 0.08) pairs. However, the MiSeq correlations were significantly affected by sample age, as four tissue samples were >10 years from collection. Correlations between FF and FFPE were significantly better in paired samples collected more recently (< 4 years) versus older pairs (> 10 years) (0.88 vs. 0.78, *p* = 0.0014) (Additional file 5 : Fig. S3). Correlations were similar between HiSeq and MiSeq when only *n* < 4 years old samples were evaluated in each platform (88% vs. 86%, *p* = 0.4051), demonstrating a high degree of similarity between gene expression profiles of matched FF and FFPE samples, regardless of platform (Additional file 5: Fig. S3).

### FF and FFPE differential transcript identification

**Fig. 4:**
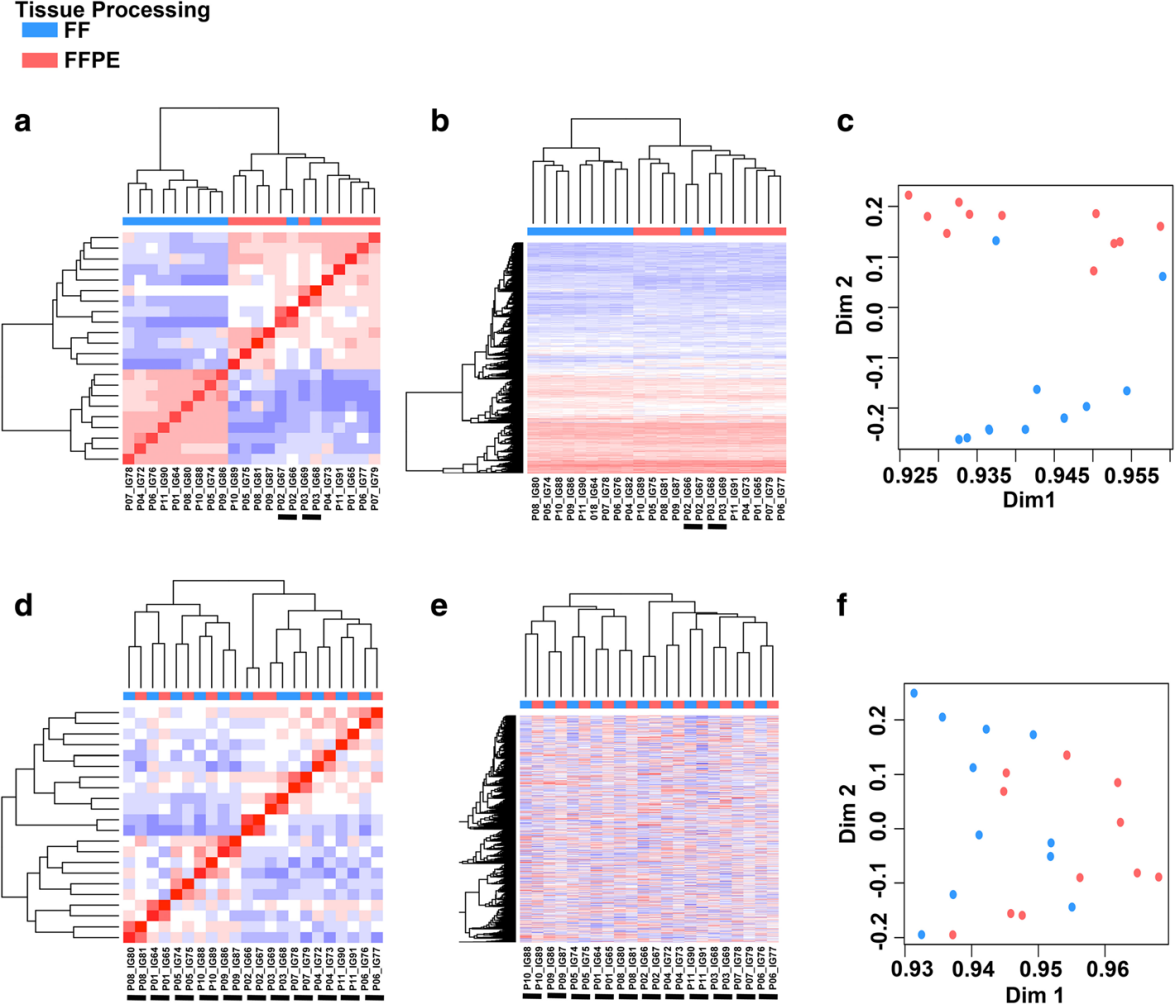
Removal of differential transcripts improves FF and FFPE gene expression correlation. *Heatmaps* display unsupervised hierarchical clustering of **a** sample-wise correlation coefficients, **b** all transcripts (*n* = 27,577) or **c** principal component analysis (PCA) of all transcripts. Following removal of differentially expressed transcripts between FF and FFPE samples, remaining transcripts (*n* = 15,624) were used to perform **d** sample-wise correlation coefficients **e** hierarchical clustering or **f** PCA. *Underlined* samples indicate clustering of paired FF and FFPE samples

To determine the similarity of expression levels for mapped transcripts between FF and FFPE samples, we performed hierarchical clustering of all the pairwise sample correlations on all transcripts (Fig. 4a and b). Only two pairs clustered together with majority of the FF and FFPE samples clustering in two groups (Fig. 4a and b). Principal component analysis (PCA) showed two groups with differential gene expression originating from FF and FFPE processing (Fig. 4c). Similar results were obtained with samples on the MiSeq platform (Additional file 6: Fig. S4). To identify transcripts that differ due to tissue processing and sequencing preparation, we performed differential gene expression analysis between FFPE and FF samples. Using a cutoff of 2-fold change (FC) and adjusted *p*-value < 0.05, we identified that 43.3% (11,953 of 27,577) of transcripts were differentially expressed on the HiSeq platform and 28% (4729 of 17,018) of transcripts differentially expressed on the MiSeq platform (Additional file 7: Table S3). The increase in differential genes present on the HiSeq platform is likely from low abundance transcripts that appear due to increased sequencing depth.

Removal of differentially expressed transcripts between FF and FFPE significantly increased the average pair-wise correlation on HiSeq (0.879 ± 0.03 to 0.957 ± 0.01, *p* = 3.7E = 6), but not the MiSeq platform (0.83 ± 0.07 to 0.89 ± 0.06,*p* = 0.11) (Additional file 4: Fig. S2B). Removal of differentially expressed genes resulted in better hierarchical clustering of each matched FF and FFPE pairs, demonstrating effective removal of transcripts that vary due to tissue processing and RNA enrichment (Fig. 4c–e).

### Differential RNA species enrichment

Since RNA was processed with different methods for FF and FFPE, we annotated transcript class and examined if sample processing preferentially enriched for different RNA species. Majority of the differential transcripts were enriched in the FFPE samples (78%, 9362 of 11,953) and most were in non-coding genes that included antisense, long non-coding RNA (lincRNA), small nuclear RNA (snRNA), small nucleolar RNAs (snoRNA), microRNA (miRNA), rRNA, 3′ noncoding (ncRNA) and pseudogene transcripts (Table 1). Protein-coding transcripts were the least differential class of transcript with 27% of protein coding differential between FF and FFPE. However, there was minimal selection bias for protein-coding genes by tissue processing as both FF (53%) and FFPE (48%) samples had similar percentages of differentially expressed protein coding transcripts. The majority of differential transcripts can be explained by differences in RNA isolation methodology. We determined that poly-A selection with FF samples enriched for coding transcripts and selected against noncoding RNA species. Similar differential transcripts were observed on the MiSeq platform (Additional file 3: Table S2).

**Table 1:** Differential transcript analysis Hi-Seq

Transcript Type	All Transcripts	Differential Transcripts (%)		FFPE (%)		FF (%)	
rRNA	44	42	(95.5%)	40	(95.2%)	2	(4.8%)
Misc RNA	437	412	(94.3%)	412	(100.0%)	0	(0.0%)
snoRNA	326	307	(94.2%)	306	(99.7%)	1	(0.3%)
snRNA	410	383	(93.4%)	383	(100.0%)	0	(0.0%)
Sense intronic	513	449	(87.5%)	448	(99.8%)	1	(0.2%)
3′ overlapping ncrna	8	7	(87.5%)	7	(100.0%)	0	(0.0%)
miRNA	232	194	(83.6%)	192	(99.0%)	2	(1.0%)
mt RNA	10	7	(70.0%)	4	(57.1%)	3	(42.9%)
Pseudogene	3661	2504	(68.4%)	2348	(93.8%)	156	(6.2%)
Antisense	2881	1949	(67.7%)	1906	(97.8%)	43	(2.2%)
Sense overlapping	116	63	(54.3%)	63	(100.0%)	0	(0.0%)
lincRNA	1992	1041	(52.3%)	1009	(96.9%)	32	(3.1%)
Processed transcript	300	138	(46.0%)	129	(93.5%)	9	(6.5%)
Polymorphic pseudogene	17	7	(41.2%)	3	(42.9%)	4	(57.1%)
Protein coding	16,630	4450	(26.8%)	2112	(47.5%)	2338	(52.5%)
Total	27,577	11,953	(43.3%)	9362	(78.3%)	2591	(21.7%)

**Fig. 5:**
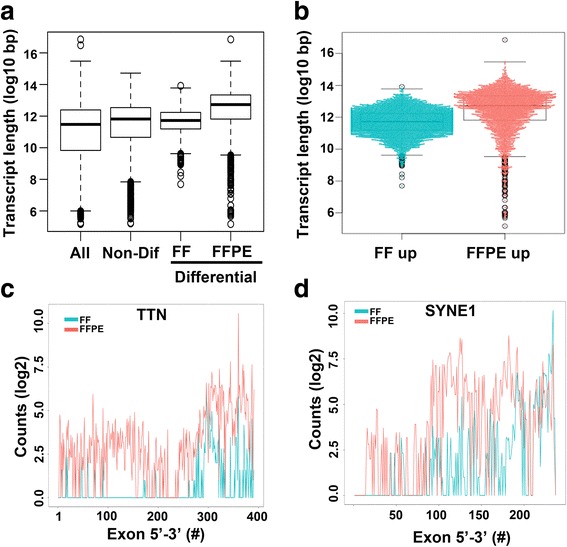
Differential transcripts are enriched for longer transcripts in FFPE compared to FF samples. **a** *Boxplot* shows transcript length (log10 bp) distribution for all protein-coding transcripts (*n* = 16,630), non-differential transcripts (*n* = 4450), transcripts enriched in FF (*n* = 2338) or FFPE (*n* = 2112). **b** *Beeswarm boxplot* shows the distribution of length for individual protein coding transcripts enriched in FF or FFPE. *Line graphs* show **c** *TTN* and **d** *SYNE1* exon level expression (count) along the transcript for in paired FF and FFPE samples

As anticipated, there was only a small increase in the number of differential protein coding transcripts in FF (53%) versus FFPE (48%). To better understand the differences in mRNA species isolated from matched FF and FFPE samples, we performed gene ontology (GO) and pathway analysis on differential protein coding transcripts that were enriched in FF or FFPE. Pathway analysis showed a typical distribution of proteins across cellular compartments (GO C5) in FF samples (Table 2) and an unexpected enrichment of transcripts coding for plasma membrane proteins in FFPE samples (Table 3). Canonical pathway (GO C2) analysis showed an enrichment of genes that encode for G-Protein Coupled Receptors (GPCR), extracellular matrix (ECM) proteins and neuronal receptors in FFPE samples. Since ECM, GPCR and membrane proteins tend to be large molecules, we examined if transcript length could account for the enrichment of plasma membrane proteins in FFPE samples. To determine if the length of protein coding transcripts is affected by FF or FFPE sample processing, we examined the base pair (bp) length of all transcripts, non-differential transcripts and differential transcripts enriched in FF or FFPE samples. The average transcript length in all protein-coding transcripts (3699 bp) was similar in non-differential transcripts (4132 bp). However differential transcripts enriched in FFPE samples were significantly longer than those enriched in FF (6309 bp vs. 3770 bp *p* < 2.2e-16) (Fig. 5a and b). To better understand this transcript length bias, we evaluated exon-level counts for two genes encoding very large transcripts, TTN and SYNE1. Examination of exon level expression showed a 3′ transcript bias for FF samples processed by poly-A selection. Our data suggest that longer transcripts are more likely to be under-represented by poly-A selection and thus may impact gene expression comparisons between FF and FFPE samples (Fig. 5c and d).

**Table 2:** Differential Pathway Enrichment in FF samples

Gene Set Name	# Genes Overlap	*p*-value	FDR *q*-value
Cellular Compartment C5			
Cytoplasm	391	1.77E-128	4.13E-126
Cytoplasmic part	265	8.87E-90	1.03E-87
Organelle part	228	2.04E-76	1.59E-74
Intracellular organelle part	227	4.69E-76	2.73E-74
Nucleus	243	3.58E-71	1.67E-69
Macromolecular complex	193	9.82E-70	3.81E-68
Mitochondrion	101	3.55E-52	1.18E-50
Protein complex	150	3.96E-48	1.15E-46
Membrane	240	1.92E-42	4.97E-41
Mitochondrial part	55	4.45E-36	1.04E-34
Canonical Pathways C2			
Huntingtons disease	71	7.32E-46	6.52E-43
TCA cycle and electron transport	63	9.81E-46	6.52E-43
Alzheimers disease	67	1.96E-44	6.53E-42
Adaptive immune system	116	1.96E-44	6.53E-42
Immune system	155	5.67E-44	1.51E-41
Oxidative phosphorylation	60	1.88E-43	4.17E-41
Parkinsons disease	59	1.08E-42	2.06E-40
Metabolism of proteins	111	2.29E-42	3.81E-40
Respiratory electron transport	50	3.40E-40	5.02E-38
Metabolism of RAN	83	2.13E-37	2.83E-35

**Table 3:** Differential Pathway Enrichment in FFPE samples

Gene Set Name	# Genes Overlap	*p*-value	FDR *q*-value
Cellular Compartment C5			
Plasma membrane	197	6.62E-61	1.03E-57
Membrane	234	3.30E-59	2.58E-56
Plasma membrane part	173	2.36E-58	1.23E-55
Membrane part	202	5.71E-53	2.23E-50
Intrinsic to membrane	177	2.20E-51	6.87E-49
Intrinsic to plasma membrane	150	2.82E-51	7.35E-49
Integral to membrane	173	1.24E-49	2.78E-47
Integral to plasma membrane	146	3.29E-49	6.43E-47
Neuroactive ligand receptor	55	1.12E-25	1.95E-23
Canonical Pathways C2			
Naba matrisome	97	6.19E-18	9.67E-16
Neuroactive ligand receptor	55	1.12E-25	1.49E-22
Matrisome	97	6.19E-18	4.11E-15
Signaling by GPCR	85	2.44E-15	9.19E-13
GPCR downstream signaling	78	2.76E-15	9.19E-13
Neuronal system	42	4.12E-15	1.10E-12
Transmembrane transport	51	1.89E-14	4.20E-12
Matrisome associated	71	1.87E-13	3.55E-11
Calcium signaling pathway	31	3.00E-13	4.98E-11
Transmission chemical synapses	28	1.48E-10	2.18E-08
GPCR ligand binding	43	3.61E-10	4.80E-08

### TNBC molecular subtyping of matched FFPE and FF RNA-seq samples

To determine if TNBC subtyping can be extended to FFPE samples profiled by RNA-seq, we performed subtyping on gene expression data derived from RNA prepared from 21 matched FF and FFPE pairs and sequenced on a HiSeq or MiSeq. Using the TNBCtype algorithm on samples sequenced on the HiSeq, we obtained subtype calls for 10 FF and 9 FFPE samples (with 9 sample pairs having calls for both FF and FFPE).

**Fig. 6:**
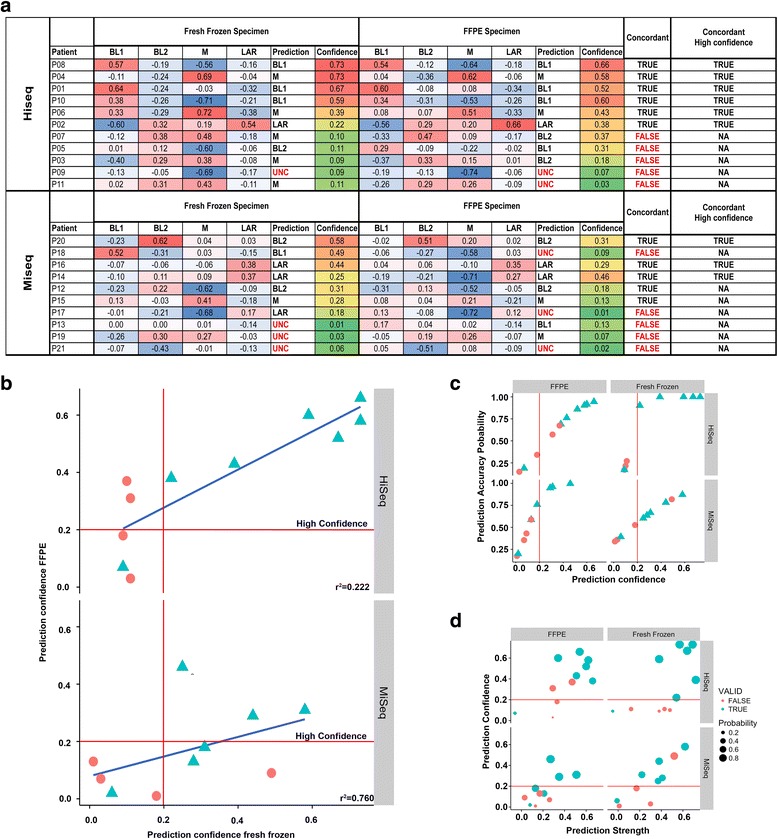
Accuracy of TNBC subtype calls between FF and FFPE depends on prediction confidence and sequencing depth. **a** Table summarizes TNBC subtype correlations, prediction calls, prediction confidence and concordance between matched FF and FFPE samples processed on the Illumina HiSeq and MiSeq. **b** *Scatter plots* show concordance (*blue*) between FF and FFPE samples run on HiSeq and MiSeq as a function of prediction confidence. **c** *Scatterplots* show the prediction confidence and prediction accuracy for FFPE (*left*) or FF (*right*) samples processed on the HiSeq (*top*) or MiSeq (*bottom*). **d** *Scatter plots* show prediction confidence and prediction strength for FFPE and FF samples processed on the HiSeq and MiSeq. Those samples with concordant subtype calls are indicated in *blue* and discordant calls in *red*

The concordance rate was 67% (6 of 9 pairs) between FF and FFPE sample sequenced on the HiSeq (Fig. 6a). Evaluation of the prediction confidence demonstrated that only those samples with high prediction confidence (those with >0.2 separation between highest and second highest subtype call), for both of the samples in a pair resulted in concordant TNBC subtypes (Fig. 6b). When samples were restricted to only high confidence prediction samples, then concordance increased to 100% (6 of 6) for the pairs (Fig. 6a). Samples sequenced on the MiSeq had greater concordance of sample calls 100% (5 of 5). However, this increased concordance was the result of decreased ability to generate distinct subtype calls with 50% of the samples unclassified. The amount of unclassified specimens sequenced on the HiSeq was 14% (3 of 22), similar to previous publications [4, 16]. This higher rate of unclassified samples on the MiSeq is reflected in the significantly lower prediction confidence scores (0.22 vs. 0.36, *p* = 0.048) than the HiSeq and weaker correlations to each of the subtypes, demonstrating increased sequencing depth can increase prediction confidence and reproducible subtyping (Fig. 6c). Correlation (prediction strength) to a particular subtype with the largest separation from other subtypes (prediction confidence) was greatest in FF samples ran on the HiSeq (Fig. 6d).

To determine if differential transcripts identified between FF and FFPE preparation of tissue affect TNBC subtype calls, we performed subtyping using only genes with similar expression between both methods, resulting in 1685 genes instead of full 2188 genes for centroid correlation. There were no differences in subtype calls between the non-differential genes (1685) and the full centroid, demonstrating the robustness of subtype prediction (Additional file 8: Table S4). TNBC subtypes could be accurately identified in FFPE samples profiled by RNA-seq and the subtype calls were not influenced by the 503 genes that are consistently differentially expressed between FFPE and FF processing.

## Discussion

Several gene signatures have been developed that provide prognostic and predictive value in breast cancer with differing tissue preparations used for development and validation. For example, the Endopredict and Oncotype DX® Recurrence Score® were originally trained, tested and validated using FFPE derived RNA, where the MammaPrint® score was trained, tested and validated using RNA isolated from fresh frozen tissues [1, 20, 21]. The breast cancer intrinsic subtypes that identify four biological and clinically distinct subtypes (Luminal A, Luminal B, HER2 and Basal-like) were developed on 1753 genes from FF samples [22], reduced to a 50 gene-set (PAM50) as an PCR-based assay using FFPE samples [23] and then adapted to a nanostring platform as the PAM50-based Prosigna test [24]. Success of predictive gene signatures is highly dependent on robust gene signatures that are reproducible across platforms, especially in the development of clinical assays. We have previously identified four tumor-derived gene expression signatures from FF samples profiled by microarray that identify distinct TNBC subtypes and have prognostic and predictive value for chemotherapy response [5, 6]. Since development, RNA-sequencing has emerged as a superior technology with increased sensitivity and limited-to-negligible background.

In order for broad clinical utility, a predictive gene signature needs to be compatible with RNA isolated from routine FFPE tissues. While others have demonstrated that reliable gene expression data can be obtained from RNA-seq performed on FFPE samples from renal cell carcinoma [8] and breast cancer [10], few prior studies have examined gene subtype accuracy between matched FF and FFPE specimens. In a comparison of an 18-gene Ras pathway signature between FF and FFPE using five technologies (Affymetrix GeneChip, NanoString, Illumina RNA-Seq, Illumina targeted RNA –seq and Illumina rRNA-depleted stranded RNA-seq), the investigators show that only the NanoString technology provides an acceptable translation of the signature from FF to FFPE and is more forgiving of poor quality RNA inputs attributed with FFPE tissues [25]. Others have shown that expression measurements of thousands of genes differed by more than two-fold in FFPE samples compared with paired FF samples, however 90% of the relative expression orderings (REOs) of gene pairs were maintained across FF and FFPE samples [26]. These studies highlight some of the difficulties in translating gene signatures between tissue preparations.

In this study, we used TCGA data to demonstrate that TNBC subtype classifications are highly conserved between microarray and RNA-seq platforms. The accuracy of subtype prediction was greater than 95% when the sample distance from the next highest subtype correlation was the greatest. Using paired matched TNBC samples we show that gene expression data obtained from matched FF and FFPE samples using RNA-seq could reliably be used to identify TNBC subtypes regardless of tissue fixation and RNA isolation methods. The concordance of TNBC subtype calls between FF and FFPE samples was dependent on prediction confidence (degree of correlation to a single subtype) and only those high confidence samples resulted in identical calls. There are several variables that can decrease prediction confidence including intratumoral and tissue heterogeneity (normal cells, sub-clonal tumor composition) in addition to biopsy sampling location. Further, we show that the accuracy of TNBC subtype prediction is substantially impacted by the age of FFPE samples, as FFPE blocks from patients isolated from older (>10 y) samples had a much lower accuracy than newer samples (<4 y). However, this time-dependent decrease in accuracy was not observed in FF samples, suggesting that age and storage of FFPE samples can alter transcript levels Therefore, researchers should remain cautious when performing retrospective TNBC molecular subtype analysis on FFPE tissues until further more in depth studies are performed on aged specimens.

Using two different sequencing platforms, we demonstrate that sequencing depth greatly improves prediction confidence and that the Illumina MiSeq platform does not provide sufficient depth for accurate prediction when evaluating the entire transcriptome. However, the MiSeq platform may be amenable to smaller hybrid based capture of the TNBC subtype signature composed solely of 2188 gene centroid or reduced subset of the signature. Using paired FF and FFPE TNBC samples we demonstrated significant differences in transcript type abundance. These were largely attributed to RNA isolation methods. While Poly-A of FF samples yields a higher degree of on-target reads compared, poly-A selection has inherent 3′ selection bias, resulting in an underrepresentation of longer protein coding transcripts compared to ribosomal depleted FFPE samples. Since poly-A selection is not possible for heavily degraded FFPE tissues, RNA isolation by ribosomal depletion must be performed and in a higher degree of off-target reads that map to intergenic or intronic regions from nascent unprocessed transcripts. In addition, ribosomal depletion needed for FFPE sample processing results in the retention of a significant amount of non-poly (A) transcripts such as miRNA, lnRNAs, snoRNAs and snRNAs. Therefore, investigators should be cautious in reducing large gene signatures to smaller clinically feasible assays and applying these tests between FF and FFPE tissues. In general, large transcripts and non-poly A RNA species should be avoided to yield similar results between tissue preparations. We anticipate these differences would be diminished when FF samples are isolated with similar methods.

Interestingly, while nearly 23% of the TNBC subtype genes were differentially expressed between matched FF and FFPE due to differing RNA enrichment methods, overall subtype calls were not affected. Since large gene signatures are not practical for clinical assay development, the TNBC subtype predictor will require gene set size reduction as to accommodate easier development of clinical diagnostic tools. This comparative study of matched FF and FFPE samples defines differentially expressed genes that should be avoided when performing future gene set reduction to smaller, clinically achievable signatures.

## Conclusions

In this study, we determined that TNBC subtype transcript classifications were reproducible between microarray and RNA-seq platforms. Further, using matched FF and FFPE samples, we demonstrated that TNBC subtypes could reliably be identified from FFPE samples with greater prediction confidence in samples less than <4 y old and samples sequenced at higher depth. TNBC subtype calls between FF and FFPE samples were identical when evaluating high-confidence samples. While there were consistent differentially expressed genes between FF and FFPE, the majority of these transcripts were the result of different RNA isolation methods. However, the knowledge of differential gene transcript representation due to RNA isolation methods can identify transcripts that should be excluded f when applying gene set size reduction strategies required for clinical implementation.

## Additional files

Additional file 1: **Table S1.** TCGA microarray and RNA-seq comparison. TCGA analysis of TNBC subtype using gene expression obtained from matched fresh-frozen specimens profiled by microarray and RNA-seq. (XLSX 106 kb)

Additional file 2: **Figure S1.** TNBC subtype correlation between RNA-seq and microarray. Correlations to TNBC subtypes do not differ with data sets generated from microarray or RNA-seq platforms. Correlations to TNBC subtypes do not differ with data sets generated from microarray or RNA-seq platforms. Scatterplots show the gene expression correlation values to TNBC subtypes (A) BL1 (B) BL2 (C) M and (D) LAR for paired samples processed by microarray and RNA-seq platforms. (TIFF 1568 kb)

Additional file 3: **Table S2.** Samples sequenced. Sample type, processing and sequencing. (XLSX 51 kb)

Additional file 4: **Figure S2.** Comparison of read alignment for FF- and FFPE-derived RNA samples sequenced on MiSeq and HiSeq. Comparison of read alignment for FF- and FFPE-derived RNA samples sequenced on MiSeq and HiSeq. Distribution of unmapped, on-target and off-target (intronic and intergenic) reads for individual FF and FFPE paired samples sequenced on the (A) HiSeq or (C) MiSeq. Boxplots shows the number of mapped reads from FF an FFPE samples sequenced on the (B) HiSeq or (D) MiSeq. (E) Boxplot show distribution of unmapped reads (%) from FF and FFPE samples obtained from new (<4 y) or old (>10 y) samples. (TIFF 833 kb)

Additional file 5: **Figure S3.** Specimen age correlation. Tumor specimen age decreases transcript correlation of sequenced FF- and FFPE-derived RNA samples. Tumor specimen age decreases transcript correlation of sequenced FF- and FFPE-derived RNA samples. Boxplots show correlation (Spearman) for (A) all transcripts or (B) non-differential transcripts between matched FF and FFPE specimens by sequencing method and age of sample (New <4y, Old >10y). (TIFF 606 kb)

Additional file 6: **Figure S4.** Differential expression of paired samples using MiSeq. Comparison of gene expression from paired FF- and FFPE-derived RNA samples before and after differential transcript removal. Comparison of gene expression from paired FF- and FFPE-derived RNA samples before and after differential transcript removal. Heatmap displays (A) unsupervised hierarchical clustering, (B) sample-wise correlation coefficients and (C) principal component analysis (PCA) for all transcripts (*n* = 27,577) or (D) hierarchical clustering (E) sample-wise correlation coefficients and (F) PCA for transcripts remaining after removal of differentially expressed genes between FF and FFPE (*n* = 15,624). (TIFF 3206 kb)

Additional file 7: **Table S3.** Differential gene expression. Differentially expressed genes between 21 paired FF- and FFPE-derived RNA samples from TNBC tumors. (XLSX 2255 kb)

Additional file 8: **Table S4.** TNBC subtype comparisons after removal of differentially expressed genes. TNBC subtype comparison between FF and FFPE samples after differential gene removal. (XLSX 21 kb)

## Abbreviations

ECM: Extracellular Matrix
FF: Fresh-frozen
FFPE: Formalin fixation and paraffin-embedding
GPCR: G-Protein Coupled Receptors
lincRNA: Long non-coding RNA
miRNA: microRNA
ncRNA: rRNA, 3′ noncoding
RNA-seq: RNA-sequencing
rRNA: ribosomal RNA
snRNA: small nuclear RNA
snoRNA: small nucleolar RNAs
TCGA: The Cancer Genome Atlas
