# Supplementary figures and images for: Comparison of triple-negative breast cancer molecular subtyping using RNA from matched fresh-frozen versus formalin-fixed paraffin-embedded tissue
Source: BMC Cancer. 2017 Apr 4;17:241. doi: 10.1186/s12885-017-3237-1 (PMC5379658; doi:10.1186/s12885-017-3237-1)

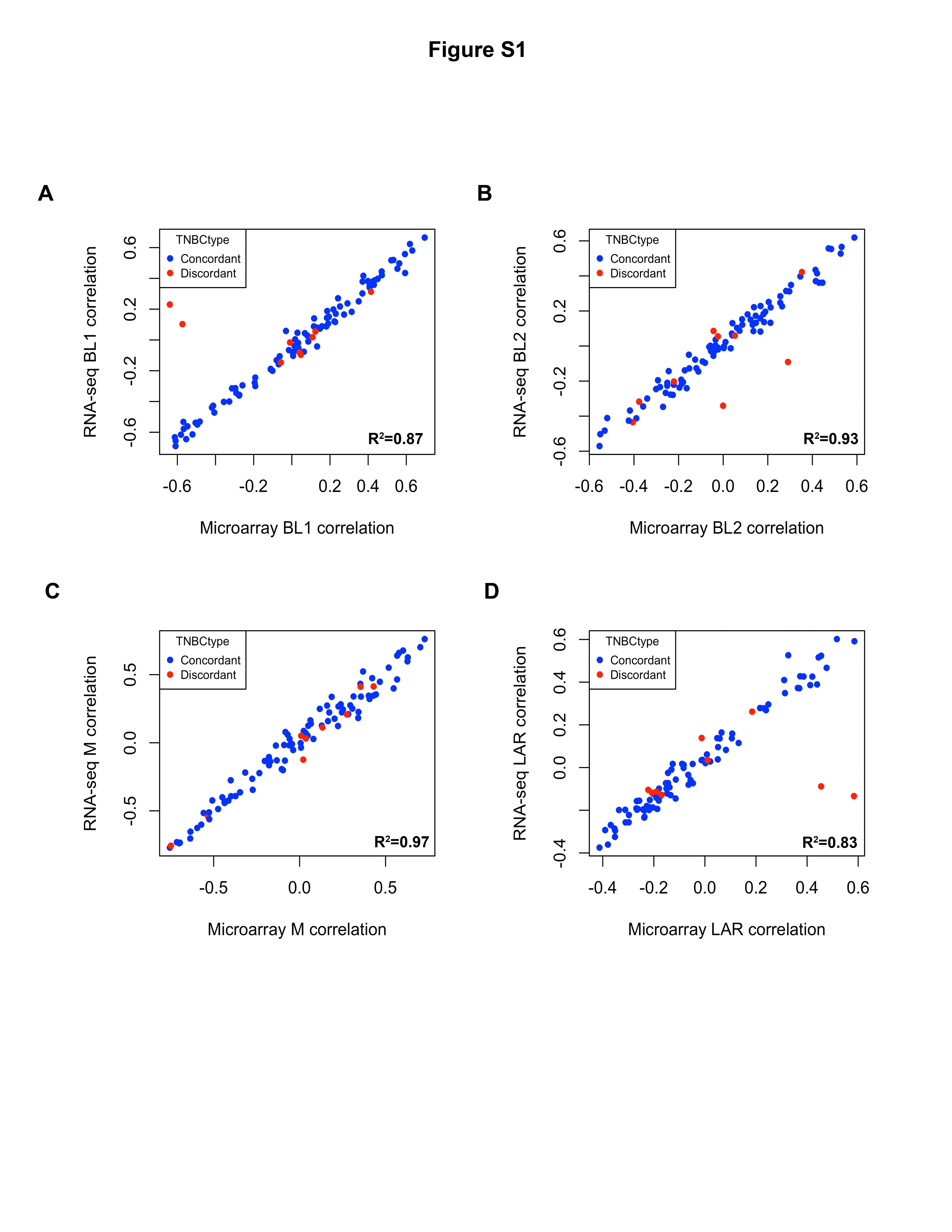

Supplement: Supplementary file 2 — Figure S1. TNBC subtype correlation between RNA-seq and microarray. Correlations to TNBC subtypes do not differ with data sets generated from microarray or RNA-seq platforms. Correlations to TNBC subtypes do not differ with data sets generated from microarray or RNA-seq platforms. Scatterplots show the gene expression correlation values to TNBC subtypes (A) BL1 (B) BL2 (C) M and (D) LAR for paired samples processed by microarray and RNA-seq platforms. (TIFF 1568 kb) [file 12885_2017_3237_MOESM2_ESM.tif]

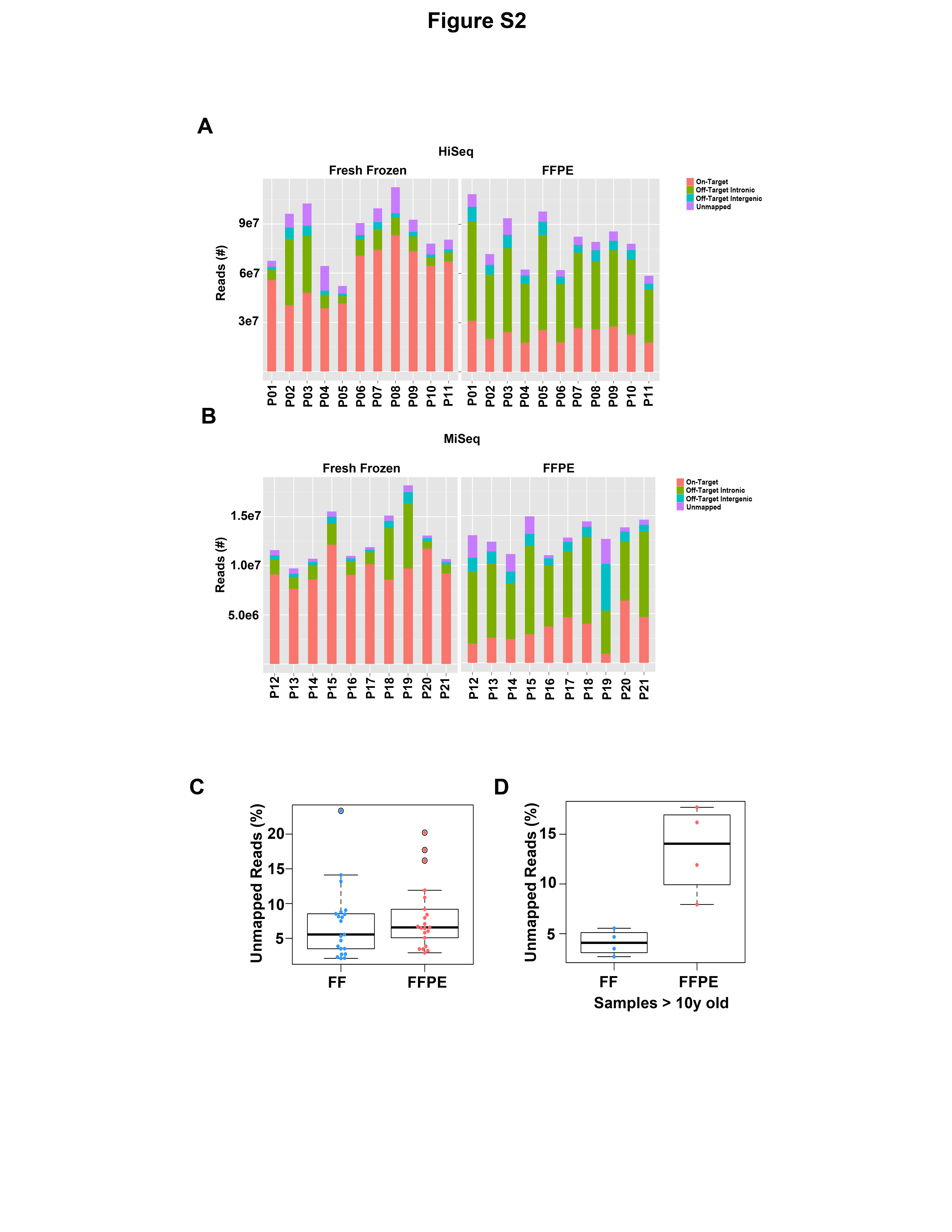

Supplement: Supplementary file 4 — Figure S2. Comparison of read alignment for FF- and FFPE-derived RNA samples sequenced on MiSeq and HiSeq. Comparison of read alignment for FF- and FFPE-derived RNA samples sequenced on MiSeq and HiSeq. Distribution of unmapped, on-target and off-target (intronic and intergenic) reads for individual FF and FFPE paired samples sequenced on the (A) HiSeq or (C) MiSeq. Boxplots shows the number of mapped reads from FF an FFPE samples sequenced on the (B) HiSeq or (D) MiSeq. (E) Boxplot show distribution of unmapped reads (%) from FF and FFPE samples obtained from new (<4 y) or old (>10 y) samples. (TIFF 833 kb) [file 12885_2017_3237_MOESM4_ESM.tif]

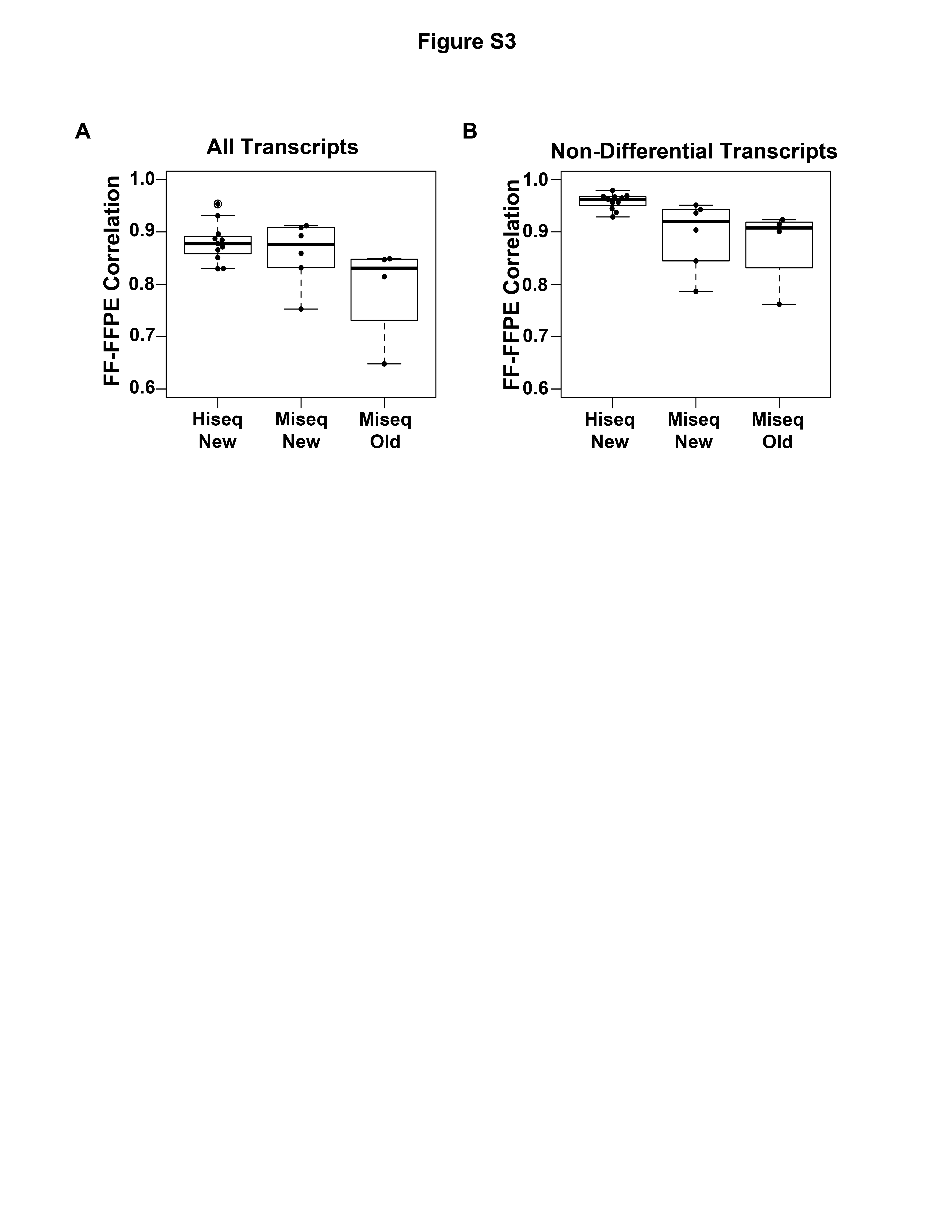

Supplement: Supplementary file 5 — Figure S3. Specimen age correlation. Tumor specimen age decreases transcript correlation of sequenced FF- and FFPE-derived RNA samples. Tumor specimen age decreases transcript correlation of sequenced FF- and FFPE-derived RNA samples. Boxplots show correlation (Spearman) for (A) all transcripts or (B) non-differential transcripts between matched FF and FFPE specimens by sequencing method and age of sample (New <4y, Old >10y). (TIFF 606 kb) [file 12885_2017_3237_MOESM5_ESM.tif]

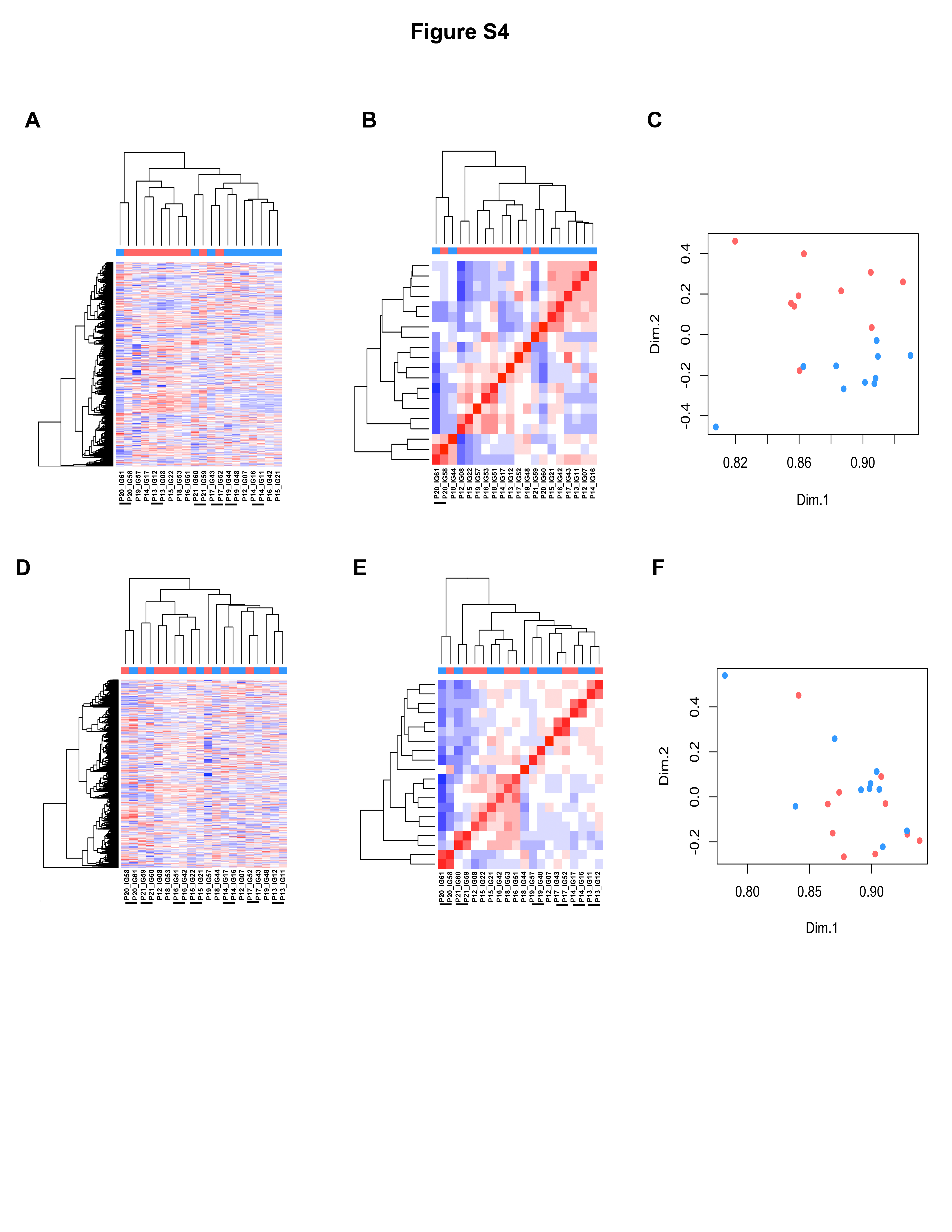

Supplement: Supplementary file 6 — Figure S4. Differential expression of paired samples using MiSeq. Comparison of gene expression from paired FF- and FFPE-derived RNA samples before and after differential transcript removal. Comparison of gene expression from paired FF- and FFPE-derived RNA samples before and after differential transcript removal. Heatmap displays (A) unsupervised hierarchical clustering, (B) sample-wise correlation coefficients and (C) principal component analysis (PCA) for all transcripts (n = 27,577) or (D) hierarchical clustering (E) sample-wise correlation coefficients and (F) PCA for transcripts remaining after removal of differentially expressed genes between FF and FFPE (n = 15,624). (TIFF 3206 kb) [file 12885_2017_3237_MOESM6_ESM.tif]
